# Impact of oral probiotics on oral health and halitosis in orthodontic patients treated with aligners or conventional fixed appliances vs placebo: two randomized clinical trials

**DOI:** 10.1093/ejo/cjag028

**Published:** 2026-05-12

**Authors:** Georgios Vasoglou, Ioulia-Maria Mylonopoulou, Gerassimos Angelopoulos, Dimitrios Kloukos, Nikolaos Pandis, Iosif Sifakakis

**Affiliations:** Department of Orthodontics, School of Dentistry, National and Kapodistrian University of Athens, 2 Thivon Street, Goudi, Athens 11527, Greece; Department of Orthodontics, School of Dentistry, National and Kapodistrian University of Athens, 2 Thivon Street, Goudi, Athens 11527, Greece; Department of Orthodontics, School of Dentistry, National and Kapodistrian University of Athens, 2 Thivon Street, Goudi, Athens 11527, Greece; Department of Orthodontics and Dentofacial Orthopedics, School of Dental Medicine/Medical Faculty, University of Bern, Freiburgstrasse 7, Bern 3010, Switzerland; Department of Orthodontics and Dentofacial Orthopedics, 251 Hellenic Air Force & VA General Hospital, 3 P. Kanellopoulou Street, Athens 11525, Greece; Department of Orthodontics and Dentofacial Orthopedics, School of Dental Medicine/Medical Faculty, University of Bern, Freiburgstrasse 7, Bern 3010, Switzerland; Department of Orthodontics, School of Dentistry, National and Kapodistrian University of Athens, 2 Thivon Street, Goudi, Athens 11527, Greece

**Keywords:** probiotics, halitosis, orthodontics, fixed appliances, aligners, volatile sulfur compounds, gingival index, plaque index

## Abstract

**Background:**

Halitosis is primarily caused by oral microbiota dysbiosis, exacerbated by orthodontic appliances that promote plaque retention. Probiotics offer a potential non-invasive strategy to rebalance oral flora and reduce volatile sulfur compounds (VSCs), but their clinical effectiveness remains largely underexplored in Orthodontics.

**Objectives:**

The aim of these trials was to determine the effect of oral probiotics containing *Lactobacillus salivarius* Oral S1 on plaque and gingival indices as well as on halitosis (via VSC) in patients treated with orthodontic brackets or aligners vs placebo.

**Methods:**

This study was ran as two separate parallel 2-arm trials (fixed appliance trial and aligner trial) in two centers. Each trial included 59 patients randomly allocated to either probiotics or placebo. The intervention was 1 month course of probiotics, compared with placebo. The primary outcome was the modified plaque index (PI-M) and the secondary outcomes were the gingival index (GI) and the VSC levels i.e. H_2_S, CH_3_SH, (CH_3_)_2_S and their sum (total VSC), all measured at baseline (T0), after 1 month of intervention (T1), and 2 months after discontinuation of the probiotics (T2).

**Results:**

In the aligner group, a significant treatment × time interaction was detected for H_2_S (*P* < .001) and total VSC (*P* < .001), indicating that the changes in these variables over time differed significantly between the treatment groups. In the fixed appliance group, a statistically significant treatment × time interaction was identified for PI-M (*P* = .01), GI (*P* < .001), H_2_S (*P* = .03), and total VSC (*P* = .03). Probiotic use was particularly effective in the latter group, with notable improvements in plaque control, gingival health, and halitosis reduction. Partial relapse occurred by T2, suggesting that benefits mostly diminish after intervention cessation.

**Conclusions:**

Administration of oral probiotics for 1 month significantly improved periodontal health and reduced halitosis in orthodontic patients, especially in those with fixed appliances.

## Introduction

Probiotics are defined as live microorganisms that when administered in adequate amounts, confer health benefits to the host, including the prevention and treatment of obesity, improvement of cognitive function, and potential applications in antitumor immunotherapy [1–3]. In the context of oral health, their utility is increasingly recognized, with evidence supporting their ability to modulate the oral microbiome, enhance the presence of beneficial bacteria, and suppress pathogenic species associated with dental caries, gingivitis, and halitosis [4–7].

Among their various applications, the role of probiotics in managing oral halitosis is particularly noteworthy. Halitosis, commonly referred to as bad breath, is primarily the result of bacterial degradation of organic substrates within the oral cavity, leading to the release of volatile sulfur compounds (VSCs) such as hydrogen sulfide (H_2_S), methyl mercaptan (CH_3_SH), and dimethyl sulfide [(CH_3_)_2_S] [8, 9]. Probiotics have demonstrated the ability to reduce the microbial load responsible for VSC production and to competitively inhibit adhesion of pathogenic species to oral surfaces [7]. Furthermore, studies have shown that specific probiotic strains can decrease tongue coating—recognized as a key reservoir for VSC-producing bacteria—thereby contributing to a reduction in halitosis [9]. The inclusion of probiotics in oral hygiene regimens, via lozenges, rinses, or other delivery systems, presents a promising alternative to conventional antimicrobial agents, since the latter are often associated with side effects such as taste alteration or mucosal irritation [10].

Notably, strains of *Lactobacillus*, *Bifidobacterium*, and *Streptococcus salivarius* K12 have demonstrated anti-inflammatory effects, suppression of biofilm formation, and significant reductions in VSC levels [10–12]. Adjunctive use of probiotics with bioactive compounds such as green tea catechins has also been shown to enhance their effects on oral malodor and plaque control [7]. By promoting a balanced oral microbiome, probiotics further contribute to systemic health by reducing inflammation associated with periodontal disease, minimizing plaque accumulation, and improving gingival health [10].

Orthodontic treatment, particularly involving fixed appliances, introduces specific challenges to oral hygiene by altering the oral microbial ecosystem. The design and placement of brackets, bands, and wires create retention sites that favor biofilm formation, thereby elevating the risk for caries, enamel demineralization, gingivitis, and halitosis [13–15]. In this context, probiotics have emerged as a potential adjunctive strategy to improve oral health outcomes.

Clinical trials involving probiotic lozenges containing strains such as *S. salivarius* M18 and K12, *Lactobacillus paracasei*, *Lactobacillus plantarum*, *Lactobacillus acidophilus*, and *Lactobacillus reuteri* have reported improvements in plaque control and gingival health among orthodontic patients [16–19]. Additionally, the combination of probiotic use with dietary changes and enhanced oral hygiene measures has been shown to exert a synergistic effect, underscoring the integration of probiotics in orthodontic care [20].

Despite these promising findings, significant variability exists across studies in terms of methodology, probiotic strains, delivery mechanisms and outcomes. Common limitations include small sample sizes [21], brief follow-up durations, and a lack of standardized dosing protocols [13, 16, 22]. Furthermore, randomized controlled trials investigating the effects of probiotics specifically in patients undergoing treatment with fixed orthodontic appliances are limited [18, 19, 21], and to date, no studies have reported on the impact of probiotics on halitosis in patients using aligners.

### Study objectives and hypothesis

The primary objective of this two-center study was to evaluate the effect of *Lactobacillus salivarius* oral probiotics (LSP) on oral hygiene plaque index (PI-M) and secondarily on gingival index (GI), as well as on halitosis [by measuring the levels of VSC i.e. H_2_S, CH_3_SH and (CH_3_)_2_S] in patients undergoing orthodontic treatment with either conventional labial fixed appliances or aligners vs placebo.

The null hypothesis of this study was that the administration of LSP does not significantly influence plaque accumulation, gingival inflammation, or halitosis in patients treated with fixed orthodontic appliances or aligners.

## Materials and methods

### Trial design and any changes after trial commencement

The trial was planned as randomized 4-arm trial and to be conducted in the Department of Orthodontics, School of Dentistry, National and Kapodistrian University of Athens (NKUA), Greece, however the COVID-19 pandemic caused serious disruptions in patient flow and recruitment for the aligner group was accomplished in the second center and therefore randomization was implemented within centers. Accordingly, the final design consisted of two independent trials conducted at two different centers.

### Participants, eligibility criteria, and setting

Participants were recruited from two sites:

- the postgraduate clinic of the Department of Orthodontics, School of Dentistry, NKUA Greece, (fixed orthodontic appliances) and
- the private orthodontic practice of one of the authors (aligners).

Inclusion criteria for all patients were the following: patients in good general health undergoing non-extraction orthodontic treatment, orthodontic treatment initiation at least 2 months prior to enrollment, with an expected treatment duration exceeding 3 months. Additionally, the fixed appliance group included adolescents aged 12–18 years, treated in both arches with conventional brackets (on premolars and anterior teeth) and bands on first permanent molars. The aligner group comprised of adult patients (≥18 years) treated with Invisalign™ covering teeth up to the second permanent molars. Patient compliance was monitored with telephone call reminders during the intervention period, at 10- and 20 days after trial initiation.

Exclusion criteria included active caries, periodontal disease, fluorosis or dental dysplasia, tooth agenesis or hypodontia, craniofacial syndromes or anomalies, psychiatric disorders, smoking or use of tobacco products, antibiotic use within the past 2 months, use of chlorhexidine or other mouthwashes within the past 3 weeks, or concurrent participation in other clinical studies [23–26].

### Interventions

Following a clinical and medical history review, eligible participants in each appliance group/center were randomized in a 1:1 ratio to either the probiotic or the placebo subgroup. The probiotic lozenges contained *L. salivarius* Oral S1 5 × 10^8^ CFU with 5 μg Vitamin D3 (Prodilac Oral, Frezyderm). The same company provided the placebo lozenges (same ingredients but without probiotics). Each patient received 30 lozenges (1 per day for 1 month) to be taken at bedtime after toothbrushing, with instructions to allow the tablet to sit on the tongue and dissolve slowly while moving it around the mouth for 3–5 min. Patients were instructed to refrain from eating or drinking for 30–60 min after taking the probiotic to ensure that the bacteria were not washed away. Oral hygiene instructions were standardized, instructing patients to brush twice daily using the same technique and the same toothpaste with a fluoride content of 1450 ppm (Frezyderm, Epismalto)—once after lunch and once before bedtime.

### Outcomes (primary and secondary)

The primary outcome was the modified Silness and Löe plaque index (PI-M) [27]. Secondary outcomes included the GI [28] and the levels of VSC.

Because the original PI does not adequately capture plaque distribution around orthodontic brackets, a modified plaque index (PI-M) was used [29]. In this index, each tooth was divided into four regions relative to the bracket: mesial, distal, gingival and incisal. Each area was scored from 0 to 3, giving a total range of 0–12 per tooth. PI-M index rating was as follows:

0: No plaque.

1: Thin plaque attached to the gingival margin and adjacent areas of the tooth. Plaque is only visible after applying a revealing solution or using a probe on the tooth surface.

2: Moderate accumulation of soft plaque deposits in the gingival sulcus or plaque on the gingival margin and adjacent areas of the tooth visible to the naked eye.

3: Abundant soft plaque deposits in the gingival sulcus or plaque on the gingival margin and adjacent areas of the tooth.

Banded molars were excluded due to difficulty in assessing plaque along gingival margins [30]. The mean plaque score per patient was calculated by averaging all measured surfaces.

For GI, plaque was assessed visually, while gingival inflammation was evaluated by gently probing the gingival margin in three locations (mesial, cervical, and distal) on the buccal surface. Unlike the PI-M, banded molars were included in the GI assessment. Mean GI scores per patient were also calculated. GI index rating was as follows:

0: Normal gums.

1: Mild inflammation, slight change in color and distinct change in texture, no bleeding upon probing.

2: Moderate inflammation, moderate redness and swelling, bleeding upon probing.

3: Severe inflammation, visible swelling, ulceration, bleeding upon probing and/or spontaneously.

VSC were measured using the OralChroma™ CHM-2 (NOVATRONIC Deutschland GmbH), a gas chromatograph that quantifies three major halitosis-related compounds: hydrogen sulfide (H_2_S), methyl mercaptan (CH_3_SH), and dimethyl sulfide [(CH_3_)_2_S]. A 1 ml disposable syringe was used to collect intraoral air after participants closed their mouths and breathed nasally for 30 s. A 0.5 ml sample was injected into the device for analysis. Assessments were performed at three time points: at baseline (T0), after 1 month of intervention (T1), and 2 months after discontinuation of the probiotics (T2). No changes were made to outcome parameters during the study.

### Sample size calculation

Previous estimates of PI-M variability in adolescents requiring orthodontic treatment were used to determine sample size (SD = 0.4) [19]: to detect a ≥30% reduction (from 1.5 to 1.1) in PI with 90% statistical power and 0.05 significance level, 22 participants per group were required. To accommodate potential dropouts, 120 patients (30 per group) were recruited. This sample size fulfills the recommendation of the American Dental Association Council on Scientific Affairs, which advises including at least 30 subjects per group for studies evaluating chemotherapeutic products for the control of gingivitis [30].

#### Interim analyses and stopping guidelines

No interim analyses were planned.

#### Randomization (random number generation, allocation concealment, implementation)

Written informed consent was obtained from the participants/participants’ parents or legal guardians prior to the randomization. Patients were randomized within each center and randomized in a 1:1 ratio to probiotic or placebo subgroups and stratified by gender. Randomization sequences—comprising 15 letters (A or B) for each sex—were generated using the List Randomizer tool (www.random.org <http://www.random.org/>). Letters were sealed in opaque, numbered envelopes (M1–M15 for males, F1–F15 for females), handled exclusively by an independent third party not involved in the study. Sequentially numbered envelopes were distributed to participants in the order of enrollment.

### Blinding

To ensure triple-blinding, lozenges were packaged identically for both probiotic and placebo groups. Placebo lozenges were identical in shape, form, smell and texture. An independent person not involved in data collection or analysis administered the interventions in both groups (fixed appliances and aligners), based on envelope assignment. Outcome evaluators were blinded to all treatment allocations (type of appliance and probiotic or placebo). Therapists, patients were blind to treatment allocations to placebo or probiotics but not to the type of orthodontic appliance. Outcome evaluators were blinded to treatment allocation.

#### Statistical analysis (primary and secondary outcomes, subgroup analyses)

To assess intra- and inter-observer reliability for PI-M and total VSC [sum of H_2_S, CH_3_SH and (CH_3_)_2_S levels], 10 patients were evaluated independently by the principal investigator and a second examiner (GA).

Descriptive statistics were computed for all outcomes by group and time point. Line plots were generated to visualize trends. Population average generalized estimating equation models with robust standard errors were fit separately per outcome and within each center. Non-parametric bootstrapping with 500 repetitions was used to estimate standard errors. Covariates included treatment group, time and their interaction and age. Statistical analyses were performed separately for the two centers. All analyses were conducted using Stata 19.5 New (StataCorp, College Station, TX, USA).

## Results

### Participant flow

**Figure 1:**
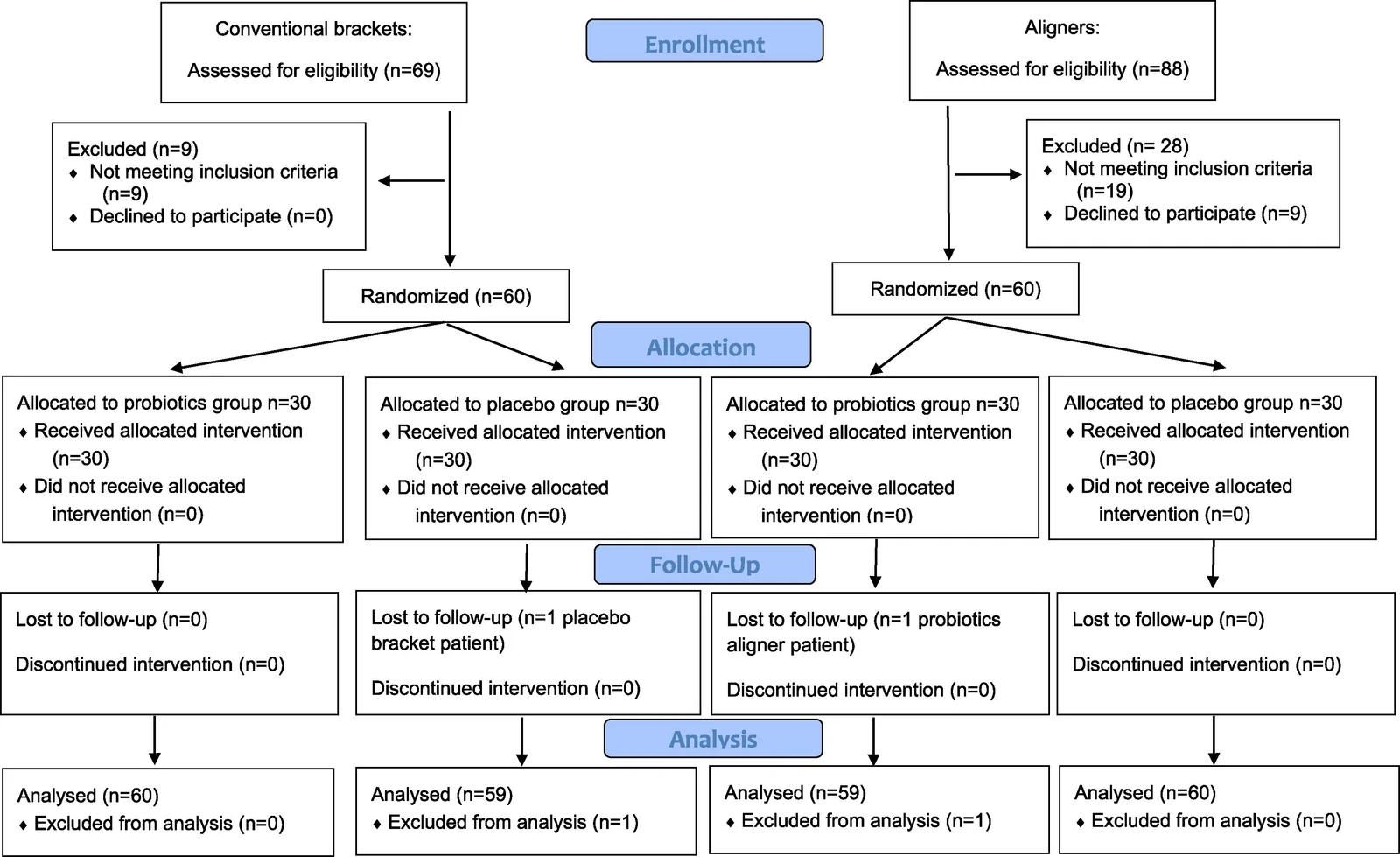
The CONSORT flow diagram.

A total of 144 patients were screened for eligibility. Recruitment continued until each of the 2 study groups included 30 per arm participants who met all inclusion criteria. Patient enrollment began in February 2021 and was completed in June 2025 (Fig. 1).

### Baseline data

Baseline characteristics of the study population are presented in Table 1. The fixed appliance group included patients aged 12–18 years, whereas the aligner group included patients aged 18–54 years.

**Table 1:** Baseline characteristics.

	Treatment group			
	A	B	C	D
	Aligner placebo (*N* = 30) median (IQR) or count	Aligner probiotics (*N* = 29) median (IQR) or count	Bracket placebo (*N* = 29) median (IQR) or count	Bracket probiotics (*N* = 30) median (IQR) or count
Age	33.80 (16.1)	34.60 (20.45)	13.65 (1.30)	13.40 (2.10)
Gender				
Female	15	14	15	15
Male	15	15	14	15
PI-M	0.12 (0.12)	0.14 (0.10)	0.30 (0.18)	0.31 (0.14)
GI	0.17 (0.03)	0.13 (0.21)	0.61 (0.09)	0.58 (0.09)
H_2_S	40.00 (48.00)	51.00 (86.00)	128.00 (91.00)	114.00 (110.00)
(CH_3_)_2_S	9.5 (25.00)	12.0 (19.00)	0.0 (17.00)	3.0 (25.00)
CH_3_SH	10.00 (27.00)	10.00 (31.00)	16.00 (16.00)	20.50 (24.00)
Total VSC sum	66.50 (73.00)	87.00 (106.00)	162.00 (113.00)	157.50 (132.00)

### Numbers analyzed for each outcome, estimation and precision, subgroup analyses

Data from 59 patients treated with fixed appliances and 59 patients treated with aligners were included in the final analysis for all outcomes. Inter- and intra-observer reliability were evaluated for the primary (PI-M) and secondary (total VSC) outcomes. Intraclass correlation coefficients (ICCs) indicated good to excellent reliability: intra-observer ICCs were 0.91 for PI-M and 0.95 for VSC, while inter-observer ICCs were 0.86 for PI-M and 0.93 for VSC. Descriptives per outcome and time point are shown in Table 2. Bracket placebo and aligner probiotics have the highest and the lowest scores, respectively, for PI-M, GI, H_2_S and total VSC at T1.

**Table 2:** Median (and interquartile range) per outcome by treatment group and time point.

		Aligner trial		Fixed appliances trial	
		Aligner placebo (*N* = 30)	Aligner probiotics (*N* = 29)	Bracket placebo (*N* = 29)	Bracket probiotics (*N* = 30)
**Outcome**	**Time**				
PI-M	T0	0.12 (0.12)	0.14 (0.10)	0.30 (0.18)	0.31 (0.14)
	T1	0.17 (0.17)	0.08 (0.12)	0.30 (0.17)	0.21 (0.15)
	T2	0.12 (0.17)	0.15 (0.10)	0.25 (0.05)	0.31 (0.09)
GI	T0	0.17 (0.031)	0.13 (0.21)	0.61 (0.09)	0.58 (0.09)
	T1	0.12 (0.08)	0.04 (0.12)	0.59 (0.07)	0.42 (0.27)
	T2	0.15 (0.21)	0.17 (0.25)	0.58 (0.10)	0.60 (0.11)
H_2_S	T0	40.00 (48.00)	51.00 (86.00)	128.00 (91.00)	114.00 (110.00)
	T1	36.00 (50.00)	12.50 (30.00)	68.00 (140.00)	59.00 (60.00)
	T2	38.00 (44.00)	11.00 (48.00)	113.00 (137.00)	82.50 (73.00)
(CH_3_)_2_S	T0	9.5 (25.00)	12.0 (19.00)	0.0 (17.00)	3.0 (25.00)
	T1	6.5 (27.00)	10.0 (18.00)	1.0 (24.00)	2.0 (21.00)
	T2	10.5 (26.00)	12.0 (18.00)	0.0 (13.00)	3.0 (27.00)
CH_3_SH	T0	10.00 (27.00)	10.00 (31.00)	16.00 (16.00)	20.50 (24.00)
	T1	12.50 (34.00)	2.00 (25.00)	18.00 (13.00)	18.00 (28.00)
	T2	14.50 (25.00)	17.00 (23.00)	19.00 (17.00)	22.50 (24.00)
Total VSC	T0	66.50 (73.00)	87.00 (106.00)	162.00 (113.00)	157.50 (132.00)
	T1	62.50 (63.00)	39.00 (44.00)	96.00 (179.00)	80.50 (90.00)
	T2	66.00 (52.00)	47.00 (51.00)	139.00 (150.00)	121.50 (101.00)

Table 3 shows the contrasts *P*-values for the main effects (treatment, time) and treatment × time interactions per outcome for the aligner group. A statistically significant treatment effect was observed for PI-M (*P* < .001), H_2_S (*P* < .001), (CH_3_)_2_S (*P* < .01), and total VSC (*P* < .001), indicating overall differences between the treatment groups for these parameters. Regarding the effect of time, significant changes were observed for GI (*P* = .02), H_2_S (*P* < .001), and total VSC (*P* < .001). A significant treatment × time interaction was detected for H_2_S (*P* < .001) and total VSC (*P* < .001), indicating that the changes in these variables over time differed significantly between the treatment groups.

**Table 3:** Overall *P*-values for the aligner group, time main effects and their interaction from the Wald test per outcome.

Outcome	Treatment group/*P*-value	Time/*P*-value	Treatment × Time interaction/*P*-value
PI-M	<.001	.18	—
GI	.30	.02	—
H_2_S	<.001	<.001	<.001
(CH_3_)_2_S	<.01	.88	—
CH_3_SH	.65	.54	—
Total VSC	<.001	<.001	<.001

Table 4 shows the *P*-values for the main effects (treatment, time) and treatment × time interactions per outcome for the fixed appliance group. A statistically significant treatment effect was observed for PI-M (*P* = .02), GI (*P* < .001), H_2_S (*P* = .001), CH_3_SH (*P* = .008), and total VSC (*P* < .01). Regarding the effect of time, significant changes were observed for PI-M (*P* = .02) and GI (*P* < .001). A statistically significant treatment × time interaction was identified for PI-M (*P* = .01), GI (*P* < .001), H_2_S (*P* = .03), and total VSC (*P* = .03), indicating that the changes in these parameters over time differed significantly between the treatment groups.

**Table 4:** Overall *P*-values for the fixed appliance group, time main effects and their interaction from the Wald test per outcome.

Outcome	Treatment group/*P*-value	Time/*P*-value	Treatment × Time interaction/*P*-value
PI-M	.02	.02	.01
GI	<.001	<.001	<.001
H_2_S	.001	.08	.03
(CH_3_)_2_S	.29	.60	—
CH_3_SH	.008	.86	—
Total VSC	<.01	.19	.03

**Figure 2:**
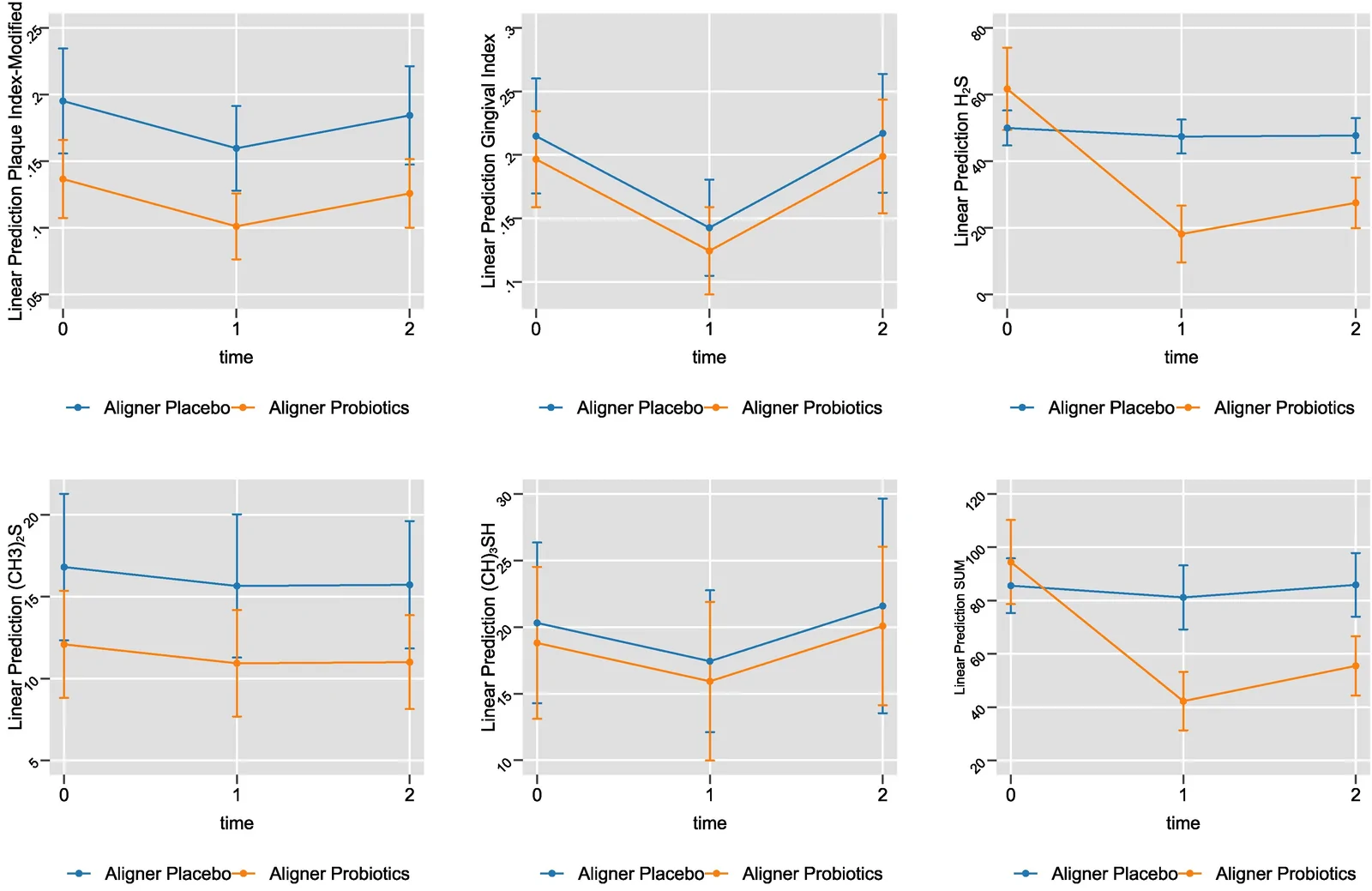
Predicted outcomes over time for the aligner group.

**Figure 3:**
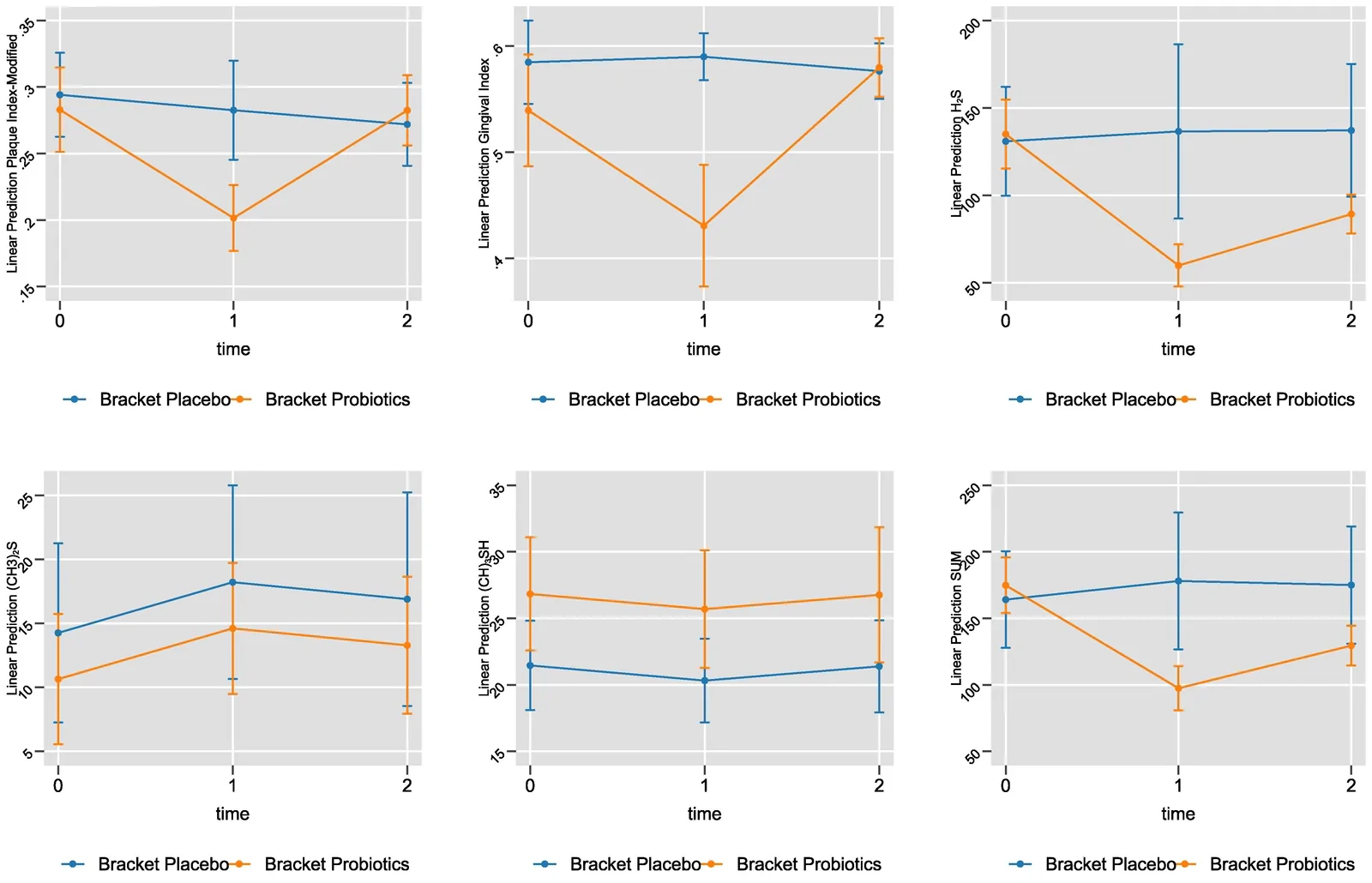
Predicted outcomes over time for the fixed appliance group.

The estimates, 95% CIs and *P*-values are shown in the Supplementary Tables S1–S6 and in the main text only the prediction plots are shown to facilitate interpretation. The linear prediction for the outcomes are depicted separately for the aligner (Fig. 2) and fixed appliance groups (Fig. 3).

## Discussion

The findings of this clinical trial support rejection of the null hypothesis. Oral probiotics containing *L. salivarius* significantly improved oral hygiene and reduced halitosis in patients undergoing orthodontic treatment, irrespective of appliance type (fixed appliances or Invisalign™ aligners). Notably, after 1 month of probiotic administration, participants demonstrated significant reductions in PI-M, GI, and halitosis, underscoring the positive impact of probiotics on oral health.

The classic Silness and Löe plaque index [27], although widely used, does not account for the plaque-retentive complexity of fixed appliances. To address this, Williams *et al*. [29] proposed the PI-M, which assesses four sites (mesial, distal, gingival, and incisal) around each bracketed tooth. This adaptation improves discrimination and sensitivity, making it more suitable for assessing plaque accumulation in orthodontic patients. Accordingly, PI-M was employed in this study to ensure precise evaluation of plaque in the fixed appliance cohort.

Fixed orthodontic appliances create plaque-retentive niches that complicate oral hygiene maintenance. Numerous studies have shown that brackets promote biofilm accumulation, resulting in elevated PI scores over time, even in patients with diligent oral hygiene practices [5, 18, 31]. In contrast, aligners are removed during eating and oral care, allowing easier access for mechanical cleaning and reducing plaque accumulation [32]. Fixed orthodontic appliances create an environment suitable for augmentation of the subgingival microbiota and are associated with poor oral hygiene [16], findings that were corroborated in our study, where patients with brackets exhibited higher PI-M scores compared with aligner users. Importantly, probiotic supplementation led to a significant reduction in PI-M for both appliance types in the present study, with particularly notable improvements in the fixed appliance group. This suggests that probiotics may be especially valuable for enhancing plaque control in patients facing greater mechanical challenges to hygiene.

Similarly, gingival inflammation is more prevalent among fixed appliance users due to increased biofilm retention. Prior studies have consistently reported higher GI scores in bracket patients compared with those using aligners [5, 21, 31], which our results affirm. However, the adjunctive use of probiotics in the present study yielded significant reductions in GI, particularly in the bracket group, supporting their role in reducing gingival inflammation, bleeding, and enhancing periodontal health.

Halitosis is a multifactorial condition which substantially impacts patients’ psychosocial well-being and interpersonal relationships. It is influenced by factors such as food retention, metabolic disorders, and respiratory tract infections. However, its predominant etiology lies in an imbalance of the oral microbiota (dysbiosis) [33]. Approximately 90% of halitosis cases originate from the oral cavity itself [12], largely due to anaerobic bacterial metabolism that produces VSC, including H_2_S and CH_3_SH [34–37]. Because halitosis is closely associated with poor oral hygiene, gingival inflammation, and periodontal disease, managing the oral microbiota is essential. Conventional antimicrobial agents, such as chlorhexidine, although effective, are often associated with adverse effects like mucosal staining and altered taste perception. In contrast, probiotics offer a natural, non-invasive alternative that modulates the microbiome and reduces VSC production [7, 10].

Halitosis may by exacerbated by fixed appliances, which promote conditions conducive to VSC production [31, 32]. Previous research suggests that aligner users typically exhibit lower VSC levels, owing to reduced plaque retention and improved hygiene access [32]. In the present study, direct comparisons of these concentrations between the two centers were not performed in order to avoid potential selection bias. Nevertheless, probiotic use significantly reduced VSC concentrations in both appliance groups, with the most pronounced effect observed in bracket patients, reinforcing the utility of probiotics in halitosis management.

Beyond *L. salivarius*, several other probiotic strains and adjunctive substances have been evaluated for their effects on halitosis. *Weissella cibaria* has demonstrated efficacy in reducing VSC production by suppressing *Fusobacterium nucleatum*, a key halitosis-associated bacterium, through hydrogen peroxide generation. The subjects were instructed to gargle twice in 24 h [33]. Another study demonstrated a decrease in VSC levels after the commencement of *S. salivarius* K12 treatment, however with limited effects on PI and GI. The subjects were tested at 7 and 14 days. Moreover, *L. reuteri* exhibits anti-inflammatory activity that supports microbial balance and indirectly improves breath odor, when tested over the span of 2 weeks [38].

In addition to probiotics, various chemical and natural agents have been investigated. Chlorhexidine and cetylpyridinium chloride effectively reduce oral malodor by disrupting biofilms but are associated with undesirable side effects such as staining and taste disturbances, after use for 4 months [39]. Fluoride compounds, including stannous fluoride, suppress acidogenic bacteria and contribute to odor control, while xylitol impedes bacterial adhesion, thereby limiting plaque and VSC production [39, 40]. Essential oils like tea tree and eucalyptus possess broad-spectrum antimicrobial properties against anaerobic pathogens implicated in halitosis [39, 41]. Zinc-based agents neutralize sulfur compounds, thereby mitigating malodor [23]. Tsironi *et al*. [42] investigated the effect of the short term (2 weeks) use of *Pistacia lentiscus* (mastic mouthwash) in orthodontic patients, reporting a reduction in hydrogen sulfide but limited impact on overall VSC levels, PI, and GI.

The probiotics preparation that was evaluated in the present study also contained vitamin D3, which may have contributed to the observed outcomes. Each lozenge (once daily) contained 5 μg D3, a dose far below the daily recommended dose. Recent research suggests that vitamin D3 supports oral health by modulating immune responses, reducing gingival inflammation, and promoting the production of antimicrobial peptides such as cathelicidins [43, 44]. Although there is no direct clinical evidence linking vitamin D3 to halitosis, it may have contributed indirectly to the reduction of bad breath through the reduction of oral inflammation and support of a balanced oral microbiome. Therefore, the presence of vitamin D3 in the probiotic supplement could have positively influenced our study results, as a synergistic effect.

In terms of clinical implications, this study suggests that incorporating oral probiotics containing *L. salivarius* Oral S1 into oral hygiene routine of orthodontic patients could be a promising strategy for enhancing plaque control, reducing gingival inflammation, and managing halitosis. However, a partial rebound of indices at T2 suggests that sustained probiotic use may be necessary to maintain these benefits over time.

Given the increasing preference for microbiome-friendly approaches, probiotics represent a sustainable adjunct to conventional oral care, particularly for individuals undergoing treatment with fixed appliances. However, most probiotic trials in the orthodontic literature were short-term (2–8 weeks), although it was proven that administration of probiotics for periods <1 month may prove insufficient for the strain to colonize because alterations in bacterial composition could not be detected. Few studies extended the tested period of the probiotics up to 3 months [2] or 17 months [45], but this long-term data was limited and did not show statistically significant clinical benefits. Across all included trials, probiotic supplementation was well tolerated, with no clinically significant adverse effects reported. Rare systemic infections with probiotics (outside orthodontic contexts) have been documented in immunocompromised patients, but this remains exceptional and not relevant to healthy orthodontic populations. Patient compliance with probiotic use was consistently good to high, reflecting favorable acceptance.

Future investigations should prioritize larger, multicentric samples and extended follow-up periods to verify the sustainability of probiotic effects.

### Limitations

This study presents some limitations. Treatments were delivered by multiple clinicians, and differences in practitioner-patient interaction could have influenced compliance. However, patient compliance was monitored with telephone call reminders.

The age distribution of the study population was determined by the demographic characteristics typical for each appliance type [46]. The fixed appliance group (12–18 years) represented the conventional orthodontic population, with a relatively homogeneous educational background, reducing behavioral variability. Younger children were excluded due to potential compliance challenges.

The aligner group comprised adults (≥18 years), as aligners are more commonly selected by this demographic. While this age discrepancy introduces potential selection bias—older individuals may exhibit better oral hygiene practices—this was mitigated by within-group comparisons between probiotics and placebo, allowing evaluation of the probiotic effect independent of age or appliance type.

Patient recruitment from two centers may have introduced selection bias, as patient demographics and behavior in academic environments may differ notably from those in private practice. Therefore, direct comparisons of the patients between the two different clinical settings were avoided.

The limitations imposed due to COVID make the comparisons between appliances less reliable. Furthermore, the exclusive inclusion of adult patients in the aligner group reduces the applicability of results to adolescent or mixed-age populations, where compliance behavior may differ significantly.

## Conclusion

This clinical trial demonstrated that oral probiotic (*L. salivarius* Oral S1) supplementation for 1 month significantly improved periodontal health and reduced halitosis in orthodontic patients undergoing treatment with fixed appliances or aligners. The probiotic intervention effectively mitigated these adverse effects, with particularly notable improvements in the fixed appliance group. The beneficial effects were evident after 1 month of use but diminished following discontinuation.

## Supplementary Material

cjag028_Supplementary_Data

## Data Availability

Data available on request.

## Trial registration

This study was registered at ClinicalTrials.gov (Identifier: NCT05651503), however as a single 4-arm trial in one center. The protocol followed the SPIRIT statement and received ethical approval from the Ethics Committee of the Data Availability Statement.
