# Supplementary material for: Impact of oral probiotics on oral health and halitosis in orthodontic patients treated with aligners or conventional fixed appliances vs placebo: two randomized clinical trials
Source: Eur J Orthod. 2026 May 12;48(3):cjag028. doi: 10.1093/ejo/cjag028 (PMC13501411; doi:10.1093/ejo/cjag028)
Supplement: Supplementary file 1 [file cjag028_supplementary_data.docx]

Supplementary Table 1. Estimates. 95% CIs and p-values for the effect of time on PI-M for both appliance types.

|  | **PI-M ALIGNER** | | |  | **PI-M FIXED** | | |
| --- | --- | --- | --- | --- | --- | --- | --- |
|  | **Coef.** | **95% CI** | **P-value** |  | **Coef.** | **95% CI** | **P-value** |
| **group** |  |  |  |  |  |  |  |
| **Placebo** | reference |  |  |  | reference |  |  |
| **Probiotics** | -0.58 | -0.09 to 0.03 | <0.001 |  | -0.01 | -0.05 το 0.03 | 0.60 |
| **time** |  |  |  |  |  |  |  |
| **0** | reference |  |  |  | reference |  |  |
| **1** | -0.03 | -0.08 to 0.005 | 0.09 |  | -0.01 | -0.06 το 0.03 | 0.65 |
| **2** | -0.01 | -0.05 to 0.03 | 0.62 |  | -0.02 | -0.06 το 0.02 | 0.33 |
| **group#time** |  |  |  |  |  |  |  |
| **Time#1** |  |  |  |  | -0.06 | -0.005 το 0.011 | 0.04 |
| **Time#2** |  |  |  |  | 0.02 | -0.005 το 0.011 | 0.48 |
| **Age** | 0.0003 | -0.001 to 0.001 | 0.69 |  | 0.003 | -0.005 το 0.011 | 0.75 |

Supplementary Table 2. Estimates. 95% CIs and p-values for the effect of time on GI for both appliance types.

|  | **GI-ALIGNER** | | |  | **GI-FIXED** | | |
| --- | --- | --- | --- | --- | --- | --- | --- |
|  | **Coef.** | **95% CI** | **P-value** |  | **Coef.** | **95% CI** | **P-value** |
| **group** |  |  |  |  |  |  |  |
| **Placebo** | reference |  |  |  | reference |  |  |
| **Probiotics** | -0.02 | -0.06 to 0.02 | 0.30 |  | -0.04 | -0.11 το 0.02 | 0.19 |
| **time** |  |  |  |  |  |  |  |
| **0** | reference |  |  |  | reference |  |  |
| **1** | -0.07 | -0.11 to -0.02 | 0.003 |  | 0.005 | -0.03 το 0.04 | 0.82 |
| **2** | -0.002 | -0.05 to 0.05 | 0.94 |  | -0.008 | -0.05 το 0.04 | 0.74 |
| **group#time** |  |  |  |  |  |  |  |
| **Time#1** |  |  |  |  | -0.11 | -0.20 το -0.02 | 0.01 |
| **Time#2** |  |  |  |  | 0.04 | -0.03 το 0.12 | 0.22 |
| **Age** | -0.002 | -0.003 to 0.0002 | 0.026 |  | -0.003 | -0.01 το 0.007 | 0.52 |

Supplementary Table 3. Estimates. 95% CIs and p-values for the effect of time on H_2_S for both appliance types.

|  | **H_2_S ALIGNER** | | |  | **H_2_S FIXED** | | |
| --- | --- | --- | --- | --- | --- | --- | --- |
|  | **Coef.** | **95% CI** | **P-value** |  | **Coef.** | **95% CI** | **P-value** |
| **group** |  |  |  |  |  |  |  |
| **Placebo** | reference |  |  |  | reference |  |  |
| **Probiotics** | 11.31 | -2.38 to 25.01 | 0.10 |  | 4.17 | -32.55 το 40.90 | 0.82 |
| **time** |  |  |  |  |  |  |  |
| **0** | reference |  |  |  | reference |  |  |
| **1** | -2.56 | -7.80 to 2.67 | 0.34 |  | 5.64 | -53.98 το 65.27 | 0.85 |
| **2** | -2.26 | -7.57 to 3.04 | 0.40 |  | 6.21 | -42.71 το 55.14 | 0.80 |
| **group#time** |  |  |  |  |  |  |  |
| **Time#1** | -40.95 | -57.91 to -23.99 | <0.001 |  | -80.74 | -144.24 το -17.24 | 0.01 |
| **Time#2** | -31.98 | -47.92 to -15.87 | <0.001 |  | -51.91 | -105.65 το 1.83 | 0.06 |
| **Age** | -0.23 | -0.58 to 0.11 | 0.19 |  | -1.05 | -7.94 το 5.84 | 0.76 |

Supplementary Table 4. Estimates. 95% CIs and p-values for the effect of time on (CH_3_)_2_S for both appliance types.

|  | **(CH_3_)_2_S ALIGNER** | | |  | **(CH_3_)_2_S FIXED** | | |
| --- | --- | --- | --- | --- | --- | --- | --- |
|  | **Coef.** | **95% CI** | **P-value** |  | **Coef.** | **95% CI** | **P-value** |
| **group** |  |  |  |  |  |  |  |
| **Placebo** | reference |  |  |  | reference |  |  |
| **Probiotics** | -4.89 | -8.40 to -1.38 | 0.01 |  | -3.47 | -9.93 το 2.98 | 0.29 |
| **time** |  |  |  |  |  |  |  |
| **0** | reference |  |  |  | reference |  |  |
| **1** | -1.15 | -6.50 to 4.19 | 0.67 |  | 3.96 | -3.76 το 11.69 | 0.31 |
| **2** | -1.08 | -5.65 to 3.48 | 0.64 |  | 2.64 | -5.94 το 11.22 | 0.55 |
| **group#time** |  |  |  |  |  |  |  |
| **Time#1** |  |  |  |  |  |  |  |
| **Time#2** |  |  |  |  |  |  |  |
| **Age** | -0.08 | -0.29 to 0.12 | 0.41 |  | -1.05 | -7.94 το 5.84 | 0.76 |

Supplementary Table 5. Estimates. 95% CIs and p-values for the effect of time on CH_3_SH for both appliance types.

|  | **CH_3_SH ALIGNER** | | |  | **CH_3_SH FIXED** | | |
| --- | --- | --- | --- | --- | --- | --- | --- |
|  | **Coef.** | **95% CI** | **P-value** |  | **Coef.** | **95% CI** | **P-value** |
| **group** |  |  |  |  |  |  |  |
| **Placebo** | reference |  |  |  | reference |  |  |
| **Probiotics** | -1.46 | -7.83 to 4.89 | 0.65 |  | 5.36 | 1.40 το 9.32 | 0.01 |
| **time** |  |  |  |  |  |  |  |
| **0** | reference |  |  |  | reference |  |  |
| **1** | -2.88 | -9.72 to 3.96 | 0.41 |  | -1.13 | -5.69 το 3.41 | 0.62 |
| **2** | 1.27 | -7.28 to 9.82 | 0.77 |  | -0.06 | -5.26 το 5.13 | 0.98 |
| **group#time** |  |  |  |  |  |  |  |
| **Time#1** |  |  |  |  |  |  |  |
| **Time#2** |  |  |  |  |  |  |  |
| **Age** | 0.02 | -0.31 to 0.35 | 0.91 |  | 0.11 | -1.70 το 1.93 | 0.90 |

Supplementary Table 6. Estimates. 95% CIs and p-values for the effect of time on total VLC for both appliance types.

|  | **TOTAL VLC ALIGNER** | | |  | **TOTAL VLC FIXED** | | |
| --- | --- | --- | --- | --- | --- | --- | --- |
|  | **Coef.** | **95% CI** | **P-value** |  | **Coef.** | **95% CI** | **P-value** |
| **group** |  |  |  |  |  |  |  |
| **Placebo** | reference |  |  |  | reference |  |  |
| **Probiotics** | 8.32 | -11.47 to 28.11 | 0.41 |  | 10.98 | -30.98 το 52.94 | 0.61 |
| **time** |  |  |  |  |  |  |  |
| **0** | reference |  |  |  | reference |  |  |
| **1** | -4.4 | -19.39 to 10.59 | 0.56 |  | 13.96 | -50.56 το 78.49 | 0.67 |
| **2** | 0.3 | -14.40 to 15.00 | 0.97 |  | 10.92 | -47.48 το 69.34 | 0.71 |
| **group#time** |  |  |  |  |  |  |  |
| **Time#1** | -47.74 | -73.35 to -22.13 | <0.001 |  | -91.36 | -161.25 το -21.47 | 0.01 |
| **Time#2** | -39.22 | -64.29 to -14.16 | 0.002 |  | -56.29 | -120.40 το 7.81 | 0.08 |
| **Age** | -0.27 | -0.81 to 0.25 | 0.31 |  | -2.23 | -9.88 το 5.41 | 0.57 |
